# Protocolo para um ensaio clínico randomizado de avaliação dos efeitos do fortalecimento extra-articular na reconstrução do ligamento cruzado anterior sobre as condições clínicas e funcionais de atletas de alto rendimento

**DOI:** 10.1055/s-0046-1822751

**Published:** 2026-04-22

**Authors:** Thiago Lemos, Conrado T. Laett, José P. G. Aramburu Filho, Geraldo R. Motta Filho, João M. Guimarães, José Leonardo R. Faria

**Affiliations:** 1 Laboratório de Pesquisa Neuromuscular e Fisiologia do Exercício, Instituto Nacional de Traumatologia e Ortopedia (INTO), Rio de Janeiro, RJ, Brasil; 2 Programa de Pós-Graduação em Ciências da Reabilitação, Centro Universitário Augusto Motta (UNISUAM), Rio de Janeiro, RJ, Brasil; 3 Hospital Central da Polícia Militar do Estado do Rio de Janeiro, Rio de Janeiro, RJ, Brasil

**Keywords:** cirurgia ortopédica, desempenho físico funcional, lesões esportivas, reabilitação, functional physical performance, orthopedic surgery, rehabilitation, sports injuries

## Abstract

**Objetivo:**

Descrever um ensaio clínico que pretende avaliar o impacto de técnicas de reforço extra-articular combinadas com a reconstrução do ligamento cruzado anterior (R-LCA) na função muscular, atividade física e retorno ao esporte em atletas de alto rendimento. A principal hipótese é que essas técnicas influenciarão diferentemente a estabilidade articular e a recuperação.

**Métodos:**

Estudo prospectivo, quase-aleatório e simples-cego pretendendo incluir 220 atletas, entre 18 e 45 anos de idade, com lesões isoladas do LCA. Os participantes serão randomizados em blocos para um dos três procedimentos: tenodese extra-articular lateral (TEL), consistindo na fixação de uma faixa da banda iliotibial próximo à origem femoral do ligamento anterolateral (LAL) com 30° de flexão do joelho para estabilidade rotatória; reconstrução do LAL com feixe único (LAL-U) utilizando um enxerto de tendão Grácil entre o Cabeça da fíbula e a tuberosidade de Gerdy; ou LAL com feixe duplo (LAL-D), empregando dois ramos do enxerto em um duplo tunel tibial entre o tuberculo de gerdy e a cabeça da fibula e fixação femoral unica na posição anatomica do LAL. As avaliações ocorrerão no pré-operatório e aos 3, 6, 9, 12 e 24 meses pós-cirúrgico, incluindo lassidão articular, morfologia do quadríceps, desempenho funcional e força isocinética. Além disso, dados autorrelatados, como o da International Knee Documentation Committee (IKDC), 36-Item Short Form Health Survey (SF-36), e a escala de Tegner também serão coletados. Modelos lineares de efeitos mistos serão usados, para análise, ajustando-se ao reparo meniscal.

**Resultados:**

Após a conclusão do estudo, esperamos identificar a técnica de reforço extra-articular mais eficaz para restaurar estabilidade e função do joelho, orientando decisões cirúrgicas. Os achados podem otimizar a reabilitação e reduzir recidivas em atletas.

**Conclusão:**

Este protocolo aborda um ensaio clínico detalhado, referente a lacunas importantes no conhecimento atual sobre as melhores abordagens cirúrgicas para R-LCA em atletas.

## Abstract (en)

**Objective:**

To describe a randomized clinical trial designed to evaluate the impact of three extra-articular reinforcement techniques combined with anterior cruciate ligament reconstruction (ACLR) on postoperative muscle function, activity levels, and return-to-sport in high-performance athletes. The primary hypothesis is that these techniques will differentially influence joint stability and recovery trajectories.

**Methods:**

A prospective, quasirandomized, single-blind trial intending to enroll 220 athletes, between 18 and 45-years-old, with isolated ACL injuries. Participants will be allocated via block randomization to one of three procedures: lateral extra-articular tenodesis (LET), involving fixation of a iliotibial band strip near the anterolateral ligament (ALL) insertion at 30° knee flexion for rotational stability; single-bundle ALL reconstruction (ALL-S), using a gracilis graft between the fibula head and Gerdy's tubercle; and double-bundle ALL (ALL-D), employing two graft limbs in a double tibial tunnel between Gerdy's tubercle and the fibular head, with a single femoral fixation in the anatomical position of the ALL. Assessments will occur preoperatively and at 3, 6, 9, 12, and 24-months postsurgery, including joint laxity, quadriceps morphology, functional performance, and knee isokinetic strength. Furthermore, self-reported outcomes, such as the International Knee Documentation Committee's (IKDC), 36-Item Short Form Health Survey (SF-36), and Tegner scale, will also be collected. Linear mixed-effects models will analyze outcomes, adjusting for meniscal repair status.

**Results:**

Upon completion, this study is expected to identify the most effective extra-articular technique for restoring knee stability and function, potentially guiding surgical decision-making. Our findings may optimize rehabilitation protocols and reduce reinjury rates in athletes.

**Conclusion:**

This protocol describes a comprehensive clinical trial that addresses important gaps in current knowledge regarding optimal surgical approaches for ACLR in athletes.

## Introdução

As lesões esportivas são geralmente decorrentes de traumas agudos ou sobrecarga crônica, variando de rupturas e luxações de ligamentos a fraturas e danos à cartilagem. As lesões no joelho são as mais prevalentes e, com frequência, representadas por rupturas do ligamento cruzado anterior (LCA), danos ao menisco e lesões na cartilagem. O joelho é particularmente vulnerável em esportes com movimentos de pivô, como futebol e basquete. Mais especificamente, as rupturas do LCA tendem a ocorrer com o joelho em extensão parcial sob estresse em valgo, 1 o que causa lassidão e déficits proprioceptivos. Danos meniscais são observados em 44 a 55% dos casos. 2

Embora o tratamento conservador seja suficiente em algumas lesões, danos estruturais comumente precisam de cirurgia para restaurar a função e facilitar o retorno às atividades esportivas. 3 A reconstrução do LCA (R-LCA) é o tratamento primário, às vezes combinada com técnicas de reforço extra-articular para maior estabilidade. Desde o surgimento da artroscopia na década de 1990, as técnicas evoluíram, utilizando enxertos dos tendões patelares, isquiotibiais ou do quadríceps. 4 A combinação de R-LCA e reparo do menisco gera melhores desfechos do que o reparo isolado. 5

Nos grupos de alto risco (ex.: atletas jovens, mulheres), o reforço extra-articular – como a tenodese de Lemaire (TEL) modificada e a reconstrução do ligamento anterolateral (LAL) – reduz as taxas de falha. Enquanto a TEL utiliza tecido da banda iliotibial, 6 a reconstrução do LAL complementa a cirurgia do LCA. 7 Estudos recentes destacam o papel das estruturas anterolaterais na estabilidade rotacional, reduzindo as taxas de reincidência de lesões. 7 No entanto, a eficácia desses métodos, acompanhados ou não por reparo do menisco, continua sob investigação.

Este estudo descreve o protocolo para um ensaio clínico randomizado concebido para avaliar os efeitos da R-LCA com diferentes técnicas extra-articulares na função muscular e nos níveis de atividade de atletas de alto rendimento. Nossa hipótese é que essas técnicas podem ter diferentes impactos na estabilidade articular, influenciando as trajetórias de recuperação. A publicação a priori deste protocolo assegura a transparência, confere proteção contra vieses de publicação ao predefinir os desfechos primários e está alinhada às melhores práticas em pesquisa clínica, conforme recomendado pelas diretrizes Standard Protocol Items: Recommendations for Interventional Trials (SPIRIT). 8

## Métodos

### Questões Éticas e Registro do Estudo Clínico

O protocolo deste estudo foi aprovado pelo Comitê de Ética local (CAAE: 84435324.0.0000.5273), em 29 de janeiro de 2025, e foi registrado no banco de dados nacional de registro de ensaios clínicos (RBR-43j2rh9), sendo a primeira versão aprovada em 8 de abril de 2025. Todos os procedimentos seguirão os princípios éticos estabelecidos na Declaração de Helsinque. Os possíveis participantes receberão explicações detalhadas sobre os procedimentos e um assistente de pesquisa fornecerá o termo de consentimento livre e esclarecido por escrito (Material Suplementar 1) para assinatura antes da inclusão no estudo. Os dados identificáveis (ex.: nomes) serão substituídos por códigos e anonimizados para publicação.

### Delineamento Experimental

Este estudo prospectivo, longitudinal, de três braços, quase-aleatório, de superioridade e simples-cego, avaliará atletas de alto rendimento com lesões do LCA em um hospital de referência ortopédica seguindo as diretrizes SPIRIT. 8

**Fig. 1:**
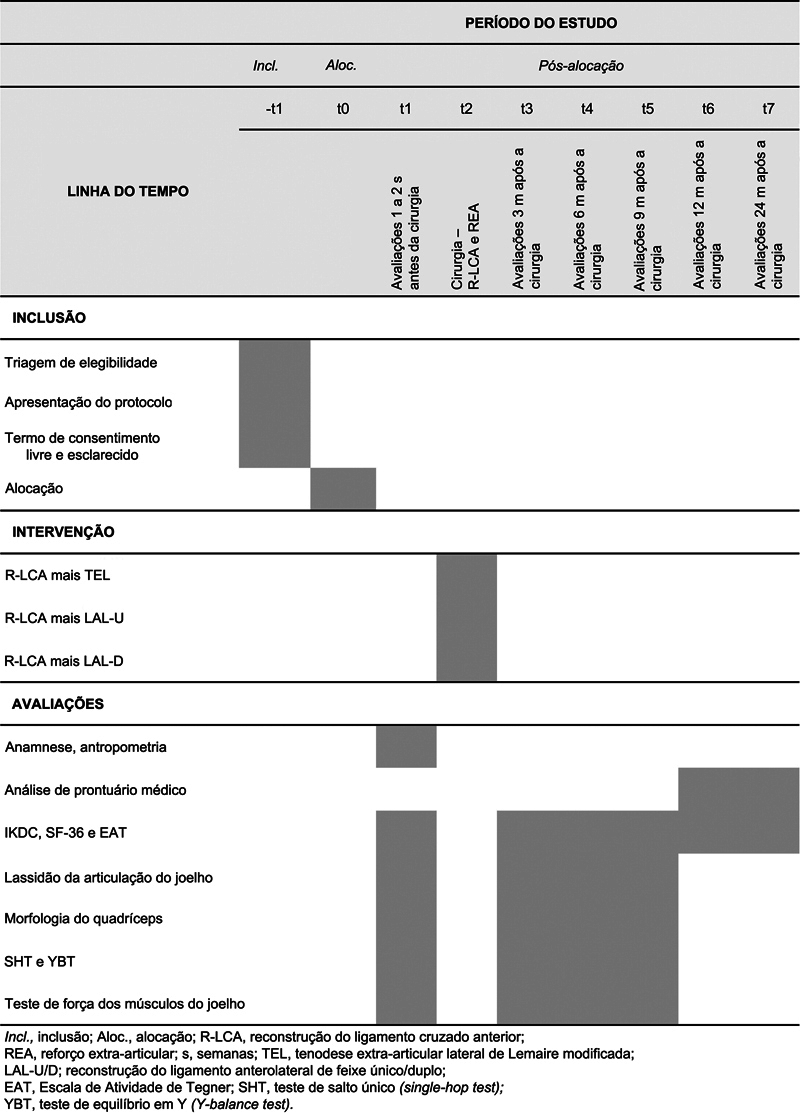
Fluxograma dos procedimentos do estudo segundo as diretrizes Standard Protocol Items: Recommendations for Interventional Trials (SPIRIT), detalhando a inclusão de pacientes, as intervenções e as avaliações em cada fase da investigação.

Os participantes serão submetidos a avaliações presenciais no início do estudo (1–2 semanas antes da cirurgia) e 3, 6 e 9 meses após a cirurgia, com coleta de dados demográficos, clínicos e funcionais (desfechos autorrelatados, lassidão do joelho, morfologia do quadríceps, força e testes de desempenho). Os testes funcionais unilaterais começarão com o membro não acometido para familiarização, e os avaliadores confirmarão previamente o conforto do participante. Cada consulta terá duração aproximada de 90 minutos. Acompanhamentos telefônicos aos 12 e 24 meses monitorarão os resultados clínicos e relatados pelos pacientes. O recrutamento, a randomização e o fluxo da intervenção estão descritos na Fig. 1 .

As emendas serão submetidas ao comitê de ética, atualizadas na plataforma ensaiosclinicos.gov.br e enviadas por e-mail aos investigadores. Os participantes serão notificados caso haja alterações nos critérios de elegibilidade ou desfechos.

### Recrutamento de Participantes

Os participantes serão recrutados da lista de espera cirúrgica do hospital ou por indicação de colegas. Os candidatos elegíveis preencherão um formulário de triagem, recebendo detalhes do estudo por telefone. Caso concordem, todos serão submetidos a avaliações médicas e aos testes para participação no estudo.

### Alocação dos Participantes

Os participantes serão alocados por um assistente de pesquisa em um dos seguintes grupos: TEL modificada, LAL-U ou -D e procedimento combinado à R-LCA. Os atletas com elegibilidade confirmada serão alocados por meio de randomização em blocos gerada por computador (proporção 1:1:1, blocos de seis) para assegurar que os grupos tenham tamanhos equilibrados ao longo de todo o período de inclusão. 9 Sequências ordenadas aleatoriamente (ex.: ABC, ACB, BAC, BCA, CAB, CBA) determinam a alocação; cada participante é submetido à próxima intervenção na sequência do seu bloco, mantendo uma distribuição igual a cada seis inclusões.

### Critérios de Elegibilidade

Os critérios de inclusão são: (1) ser atleta federado e ter participado de competições oficiais nos últimos 2 anos; (2) ter entre 18 e 45 anos de idade; (3) ter diagnóstico de lesão isolada do LCA sem lesão concomitante de outros ligamentos do joelho; e (4) sem cirurgia prévia na articulação acometida. Os critérios de exclusão são infecção pós-operatória, impossibilidade de comparecer às avaliações de acompanhamento ou recusa em participar das avaliações agendadas.

### Intervenções

Na TEL modificada, coleta-se uma faixa da banda iliotibial, que é passada profundamente ao ligamento colateral lateral e fixada próxima à inserção femoral do LAL, com o joelho em 30° de flexão e rotação neutra, para proporcionar estabilidade rotacional e evitar restrição excessiva. 6

O tendão do semitendíneo foi preparado em configureção tripla e associado ao tendão do grácil de formato único, resultando em um enxerto com quatro bandas na porção intra-articular correspondente ao LCA. A banda mais longa, proveniente do grácil, foi utilizada para a reconstrução do LAL. O tunel femoral é confeccionado com guia de fora para dentro, deixando o tunel anatomico para o LCA intra-articular e anatomico para o LAL extra-articular 5 mm posterior e proximal ao epicondilo lateral. Na tibia o enxerto do gracil é distalizado passando superficial ao LCL e profundo ao trato iliotibial, sendo inserido na tibia na posição entre o tuberculo de gerdy e a cabeça da fibula, à 1 cm distal a articulação. 10 Por fim, a técnica LAL-D utiliza os mesmos enxertos que a técnica LAL-U, com a mesma fixação femoral citada previamente. A fixação tibial é realizada atraves de um duplo tunel que se comunica, posicionado entre o tuberculo de gerdy e a cabeça da fibula 00E0; 1 cm da interlinha articular. Formando uma configureção de dupla banda do LAL, com intuito de mimetizar a estrutura nativa em formato do leque do ligamento. 7

### Desfechos

As avaliações dos participantes ocorrerão em duas fases: pré-operatória (1–2 semanas antes da cirurgia) e pós-operatória, em consultas ambulatoriais aos 3, 6 e 9 meses. As avaliações incluirão revisão do histórico médico geral, medidas autorrelatadas de funcionalidade, métricas de qualidade de vida, níveis de atividade física, assim como medidas clínicas de lassidão articular, morfologia do quadríceps e força de extensão do joelho. Os acompanhamentos telefônicos aos 12 e 24 meses coletarão dados autorrelatados de funcionalidade, qualidade de vida e níveis de atividade.

Quatro questionários validados serão empregados: o Formulário Subjetivo de Joelho da International Knee Documentation Committee (IKDC) para determinação de funcionalidade, que demonstrou propriedades psicométricas superiores às alternativas; 11 e o Medical Outcomes Short-Form Health Survey (SF-36) de qualidade de vida, que estabeleceu referências normativas para a população local e comprovou sua aplicabilidade em pacientes ortopédicos. 12 Por fim, a Escala de Atividade de Tegner para avaliação dos níveis de atividade anteriores à lesão e atuais. 13 Um questionário personalizado de tratamento registrará os detalhes clínicos e fisioterapêuticos. As respostas serão registradas eletronicamente em formulários anonimizados para assegurar a confidencialidade.

As análises de lassidão articular serão realizadas com o artrômetro KT-2000 (MEDmetric Corp.) de acordo com as especificações do fabricante. 14 Três medidas consecutivas de cada membro serão obtidas e os valores médios serão utilizados nas avaliações subsequentes.

A morfologia do quadríceps será determinada por meio de ultrassonografia padronizada adquirida com o sistema Mindray Z6 (Shenzhen Mindray Bio-Medical Electronics Co. Ltd.), com um transdutor linear de 40 mm (7–15 MHz). Imagens panorâmicas transversais dos músculos vasto lateral, reto femoral e vasto intermédio serão capturadas. 15 A análise subsequente das imagens, utilizando o software ImageJ (gratuito e de código livre), quantificará a espessura muscular e a intensidade do eco.

O desempenho funcional será avaliado por meio do teste de salto único (SHT, single-hop test em inglês) e do teste de equilíbrio em Y (YBT, Y-balance test em inglês) para determinar a força dos membros inferiores e o controle postural dinâmico 16 17 de acordo com protocolos padronizados. No SHT, os participantes ficarão em pé atrás de uma linha demarcada, saltarão horizontalmente sobre uma perna e aterrissarão sem perder o equilíbrio. A média de três tentativas será analisada. No YBT, os participantes ficarão descalços sobre uma perna no centro de uma marcação em forma de Y, estendendo a outra perna em três direções (anterior, posteromedial e posterolateral), tocando levemente a linha antes de retornar. A média de três tentativas por direção será calculada.

A força do joelho será medida com um dinamômetro isocinético (Humac Norm II, Computer Sports Medicine Inc.) a 60°/s enquanto os participantes repetem movimentos de extensão e flexão do joelho com esforço máximo. 15 18 Ao longo do teste, o avaliador monitorará e documentará qualquer dor relatada, assim como os valores de pico de torque e quaisquer observações de dor.

Os dados demográficos e clínicos (altura, massa corporal, tabagismo, comorbidades) serão obtidos de prontuários médicos ou por meio de entrevistas estruturadas. Todos os procedimentos asseguram a privacidade dos participantes durante a coleta dos desfechos clínicos e funcionais relevantes.

Lembretes de acompanhamento por telefone/e-mail e agendamentos flexíveis serão utilizados para promover a retenção de participantes. Os dados de desfechos (IKDC, SF-36, escalas de Tegner e taxas de reincidência de lesão) serão coletados por telefone caso os pacientes interrompam a avaliação física, mas concordem em participar do acompanhamento.

### Tamanho da Amostra

O tamanho da amostra foi calculado para uma análise de modelo misto com comparação de três grupos (TEL, LAL-U e LAL-D) em seis momentos (pré-intervenção e 3, 6, 9, 12 e 24 meses pós-intervenção), com ajuste segundo o reparo meniscal. Com base em um modelo de análise de covariância (ANCOVA), com tamanho de efeito de f = 0,25, alfa = 5% e poder de 80% (G*Power 3.1.9.2, Heinrich-Heine-Universität Düsseldorf), a estimativa foi de N = 220. Os dados faltantes serão tratados por meio de métodos de imputação.

### Cegamento

Este ensaio clínico simples-cego assegura o cegamento apenas para o pesquisador responsável pela coleta e análise dos dados, enquanto cirurgiões, participantes e cuidadores permanecem cientes da alocação dos tratamentos para manutenção da objetividade.

### Análise Estatística

A análise estatística utilizará modelos lineares de efeitos mistos para avaliação do grupo de intervenção, o tempo e seus efeitos de interação, com ajuste segundo o reparo meniscal como covariável. O modelo inclui fatores fixos para grupo (0 = TEL, 1 = LAL-U, 2 = LAL-D), tempo (pré-intervenção, 3, 6, 9, 12 e 24 meses pós-intervenção) e condição do menisco (0 = ausente e 1 = presente), além de suas interações e interceptos aleatórios para cada participante. As premissas do modelo (normalidade e homocedasticidade) serão verificadas por meio de gráficos Q-Q e testes de Levene.

As comparações post hoc das médias marginais aplicarão a correção de Bonferroni entre os grupos e os tempos. Todas as análises ocorrerão em ambiente Python (Python Software Foundation) utilizando o pacote *statsmodels* .

### Manejo e Monitoramento de Dados

A entrada de dados será realizada duas vezes por pesquisadores independentes para minimizar erros e, a seguir, verificações contínuas serão conduzidas para identificação de valores discrepantes. Os formulários eletrônicos serão armazenados em bancos de dados restritos, acessíveis ao pesquisador principal, aos estatísticos e às autoridades regulatórias. A equipe de pesquisa revisará anualmente as questões de segurança e adesão, monitorando eventos adversos e desvios de protocolo. Análises parciais estão planejadas para 50% dos participantes (N = 110). Quaisquer eventos adversos serão registrados e relatados ao comitê de ética utilizando formulários padronizados.

## Discussão

Este protocolo de estudo apresenta um ensaio clínico prospectivo concebido para avaliar e comparar duas técnicas cirúrgicas de R-LCA combinadas ao reforço extra-articular em atletas de alto rendimento. A investigação da TEL modificada 6 e LAL-U e LAL-D 10 fornecerá informações importantes sobre como otimizar a recuperação funcional e o retorno ao esporte após uma lesão do LCA.

Os achados esperados deste ensaio clínico podem contribuir significativamente para o entendimento atual de como diferentes abordagens cirúrgicas influenciam os desfechos pós-operatórios em populações de atletas. Para assegurar a ampla divulgação, os resultados serão publicados em acesso aberto e compartilhados nas redes sociais. O protocolo completo, o código estatístico e os dados dos participantes serão disponibilizados mediante solicitação razoável.

A relevância clínica deste estudo reside em seu potencial para esclarecer qual técnica de reforço extra-articular pode ser mais adequada para diferentes perfis de atletas. Com base em evidências biomecânicas existentes, hipotetizamos que a TEL pode ter vantagens na estabilidade dinâmica nos primeiros estágios por restaurar imediatamente a estabilidade mecânica na translação anterior e rotação interna do joelho. 19 Em contrapartida, hipotetiza-se que todas as técnicas de reconstrução (LAL-U e LAL-D) gerem desfechos superiores em longo prazo na restauração da força e no desempenho funcional, replicando com maior precisão a biomecânica nativa do joelho. 7 Esses achados esperados podem ajudar a tomada de decisões mais informadas ao escolher técnicas cirúrgicas com base nas demandas específicas do esporte e nos objetivos de reabilitação de um atleta.

Este protocolo se baseia e amplia a literatura atual de diversas maneiras importantes. Embora estudos anteriores tenham estabelecido os benefícios de procedimentos extra-articulares na redução da lassidão rotacional e das taxas de falha do enxerto em comparação à R-LCA isolada, ainda há poucas evidências de alta qualidade que confrontem diretamente diferentes técnicas de reforço em populações de atletas. 20 A inclusão de avaliações funcionais abrangentes em múltiplos momentos permitirá o exame detalhado das trajetórias de recuperação, podendo identificar janelas críticas para intervenções direcionadas de reabilitação. Além disso, ao considerar sistematicamente o estado do menisco como covariável, este estudo visa contribuir para a resolução de debates atuais sobre como o reparo meniscal concomitante influencia os tempos de recuperação pós-operatória.

A abordagem metodológica incorpora diversas características concebidas para assegurar resultados robustos e confiáveis. O delineamento longitudinal, com avaliações agendadas em vários momentos pós-operatórios, permitirá captar tanto a recuperação funcional em curto prazo quanto os desfechos em longo prazo relevantes para o desempenho atlético. A combinação de medidas de desfecho relatadas pelo paciente com testes objetivos de desempenho físico proporciona uma avaliação multidimensional da recuperação, refletindo as perspectivas clínicas e funcionais.

Protocolos cirúrgicos e diretrizes de reabilitação padronizados foram implementados para minimizar a variabilidade na execução da técnica e nos cuidados pós-operatórios entre os grupos de tratamento. Embora o cegamento completo dos participantes e cirurgiões não seja viável devido à natureza das intervenções, a utilização de avaliadores de desfecho e estatísticos que desconheçam a alocação dos participantes aos grupos ajudará a manter a objetividade na coleta e análise dos dados.

Diversas limitações devem ser consideradas na interpretação dos resultados futuros. O delineamento quase aleatório, embora prático para estudos cirúrgicos, pode introduzir um viés de seleção que talvez influencie os desfechos. Para mitigar esse viés, características basais detalhadas serão coletadas e consideradas na análise estatística. O foco em atletas de alto rendimento, embora apropriado para o estudo de indivíduos com demanda física elevada, pode limitar a generalização dos resultados para atletas recreativos ou populações de não atletas. O extenso período de acompanhamento necessário para avaliação de desfechos em longo prazo pode levar à perda de participantes, embora estratégias como acompanhamento telefônico e incentivos tenham sido incorporadas para aumentar a retenção.

## Conclusão

Este protocolo descreve um ensaio clínico abrangente que aborda lacunas importantes no conhecimento atual sobre as abordagens cirúrgicas ideais para a R-LCA em atletas. Ao comparar sistematicamente as técnicas TEL, LAL-U e LAL-D, o estudo visa gerar evidências que possam orientar a tomada de decisões clínicas e, se possível, estabelecer abordagens mais padronizadas para o tratamento de lesões do LCA em atletas de alto rendimento. Após a conclusão, os resultados poderão influenciar a escolha da técnica cirúrgica, os protocolos de reabilitação e, em última análise, melhorar os desfechos de atletas que retornam a atividades esportivas complexas.

## References

[1] Lucarno S Zago M Buckthorpe M Systematic Video Analysis of Anterior Cruciate Ligament Injuries in Professional Female Soccer Players Am J Sports Med 2021 49 07 1794 1802 10.1177/03635465211008169 33989090

[2] Bellabarba C Bush-Joseph C A Bach B R Jr Patterns of meniscal injury in the anterior cruciate-deficient knee: a review of the literature Am J Orthop 1997 26 01 18 23 9021030

[3] Buerba R A Zaffagnini S Kuroda R Musahl V ACL reconstruction in the professional or elite athlete: state of the art J ISAKOS 2021 6 04 226 236 10.1136/jisakos-2020-000456 34272299

[4] Butt U M Khan Z A Amin A Shah I A Iqbal J Khan Z Peroneus Longus Tendon Harvesting for Anterior Cruciate Ligament Reconstruction JBJS Essent Surg Tech 2022 12 02 e20.00053 10.2106/JBJS.ST.20.00053 PMC9889288 36741045

[5] Espejo-Reina A Serrano-Fernández J M Martín-Castilla B Estades-Rubio F J Briggs K K Espejo-Baena A Outcomes after repair of chronic bucket-handle tears of medial meniscus Arthroscopy 2014 30 04 492 496 10.1016/j.arthro.2013.12.020 24680309

[6] Pavão D M Cruz R S Faria JLRd Sousa EBd Barretto J M Modified Lemaire Lateral Tenodesis Associated With an Intra-articular Reconstruction Technique With Bone-Tendon-Bone Graft Using an Adjustable Fixation Mechanism Arthrosc Tech 2019 8 07 e733 e740 10.1016/j.eats.2019.03.009 31485400 PMC6713996

[7] Sonnery-Cottet B Daggett M Helito C P Fayard J M Thaunat M Combined Anterior Cruciate Ligament and Anterolateral Ligament Reconstruction Arthrosc Tech 2016 5 06 e1253 e1259 10.1016/j.eats.2016.08.003 28149722 PMC5263705

[8] Chan A W Tetzlaff J M Altman D G SPIRIT 2013 statement: defining standard protocol items for clinical trials Ann Intern Med 2013 158 03 200 207 10.7326/0003-4819-158-3-201302050-00583 23295957 PMC5114123

[9] Portney L G Foundations of Clinical Research: Applications to Evidence-Based Practice. 4th ed. Philadelphia F.A. Davis 2020

[10] Helito C P Bonadio M B Gobbi R G Combined Intra- and Extra-articular Reconstruction of the Anterior Cruciate Ligament: The Reconstruction of the Knee Anterolateral Ligament Arthrosc Tech 2015 4 03 e239 e244 10.1016/j.eats.2015.02.006 26258037 PMC4523866

[11] Metsavaht L Leporace G Riberto M de Mello Sposito MM Batista L A Translation and cross-cultural adaptation of the Brazilian version of the International Knee Documentation Committee Subjective Knee Form: validity and reproducibility Am J Sports Med 2010 38 09 1894 1899 10.1177/0363546510365314 20472755

[12] Laguardia J Campos M R Travassos C Najar A L Anjos L AD Vasconcellos M M Brazilian normative data for the Short Form 36 questionnaire, version 2 Rev Bras Epidemiol 2013 16 04 889 897 10.1590/S1415-790x2013000400009 24896594

[13] Tegner Y Lysholm J Rating systems in the evaluation of knee ligament injuries Clin Orthop Relat Res 1985 198 43 49 10.1097/00003086-198509000-00007 4028566

[14] Niu X Mai H Wu T Reliability of a Novel Automatic Knee Arthrometer for Measuring Knee Laxity After Anterior Cruciate Ligament Ruptures Orthop J Sports Med 2022 10 02:23259671211051301 10.1177/23259671211051301 PMC8855393 35187181

[15] Laett C T Cossich V Goes R A Gavilão U Rites A Oliveira CGd Relationship between vastus lateralis muscle ultrasound echography, knee extensors rate of torque development, and jump height in professional soccer athletes Sport Sci Health 2021 17 06 299 306 10.1007/s11332-020-00681-z

[16] Davies W T Myer G D Read P J Is It Time We Better Understood the Tests We are Using for Return to Sport Decision Making Following ACL Reconstruction? A Critical Review of the Hop Tests Sports Med 2020 50 03 485 495 10.1007/s40279-019-01221-7 31745732 PMC7018781

[17] Plisky P J Rauh M J Kaminski T W Underwood F B Star Excursion Balance Test as a predictor of lower extremity injury in high school basketball players J Orthop Sports Phys Ther 2006 36 12 911 919 10.2519/jospt.2006.2244 17193868

[18] Albarello J CS Laett C T De Palma A MS Associated ACL Reconstruction and Meniscal Repair do not Affect the Evolution of Isokinetic Parameters in Professional Athletes: A Prospective Study with a One-Year Follow-Up Muscles Ligaments Tendons J 2024 14 03 450 457 10.32098/mltj.03.2024.08

[19] Delaloye J R Hartog C Blatter S Anterolateral Ligament Reconstruction and Modified Lemaire Lateral Extra-Articular Tenodesis Similarly Improve Knee Stability After Anterior Cruciate Ligament Reconstruction: A Biomechanical Study Arthroscopy 2020 36 07 1942 1950 10.1016/j.arthro.2020.03.027 32251683

[20] Damayanthi E D Kholinne E Singjie L C Sakti M Anesstesia I J Combined Anterior Cruciate Ligament Reconstruction (ACLR) and Lateral Extra-articular Tenodesis through the Modified Lemaire Technique versus Isolated ACLR: A Meta-analysis of Clinical Outcomes Rev Bras Ortop 2024 59 02 e180 e188 10.1055/s-0044-1785492 PMC11006520 38606123

